# Supplementary material for: Comparative Cytological and Gene Expression Analysis Reveals That a Common Wild Rice Inbred Line Showed Stronger Drought Tolerance Compared with the Cultivar Rice
Source: Int J Mol Sci. 2024 Jun 28;25(13):7134. doi: 10.3390/ijms25137134 (PMC11241580; doi:10.3390/ijms25137134)
Supplement: Supplementary file 1 [file ijms-25-07134-s001.zip › ijms-3027527-supplementary/Supplementary Files/Supplementary Figures.pdf]

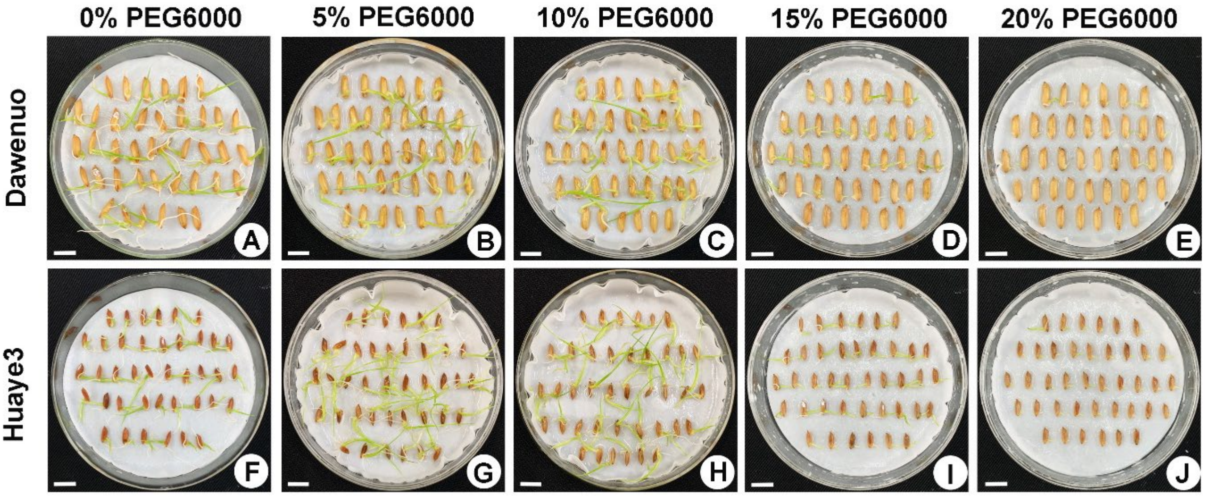

Figure S1. Comparison of seed germination in two materials under different concentrations of PEG6000 on the 7th day

(A) The seed germination of Dawennuo on the 7th day with no PEG6000, CK. (B) The seed germination of Dawennuo on the 7th day under 5% PEG6000. (C) The seed germination of Dawennuo on the 7th day under 10% PEG6000. (D) The seed germination of Dawennuo on the 7th day under 15% PEG6000. (E) The seed germination of Dawennuo on the 7th day under 20% PEG6000. (F) The seed germination of Huaye3 on the 7th day with no PEG6000, CK. (G) The seed germination of Huaye3 on the 7th day under 5% PEG6000. (H) The seed germination of Huaye3 on the 7th day under 10% PEG6000. (I) The seed germination of Huaye3 on the 7th day under 15% PEG6000. (J) The seed germination of Huaye3 on the 7th day under 20% PEG6000. Bars=1 cm.

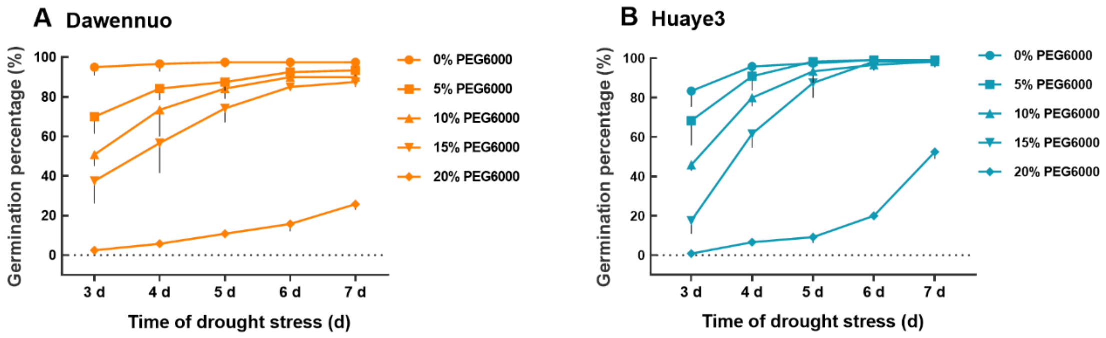

Figure S2. Comparison of seed germination trends of Dawennuo and Huaye 3 under different concentrations of PEG6000 stress

(A) Germination of Dawennuo under different concentrations of PEG6000 stress from the 3rd day to the 7th day. (B) Germination of Huaye3 under different concentrations of PEG6000 stress from the 3rd day to the 7th day. The individual gray line segments indicated the standard errors.

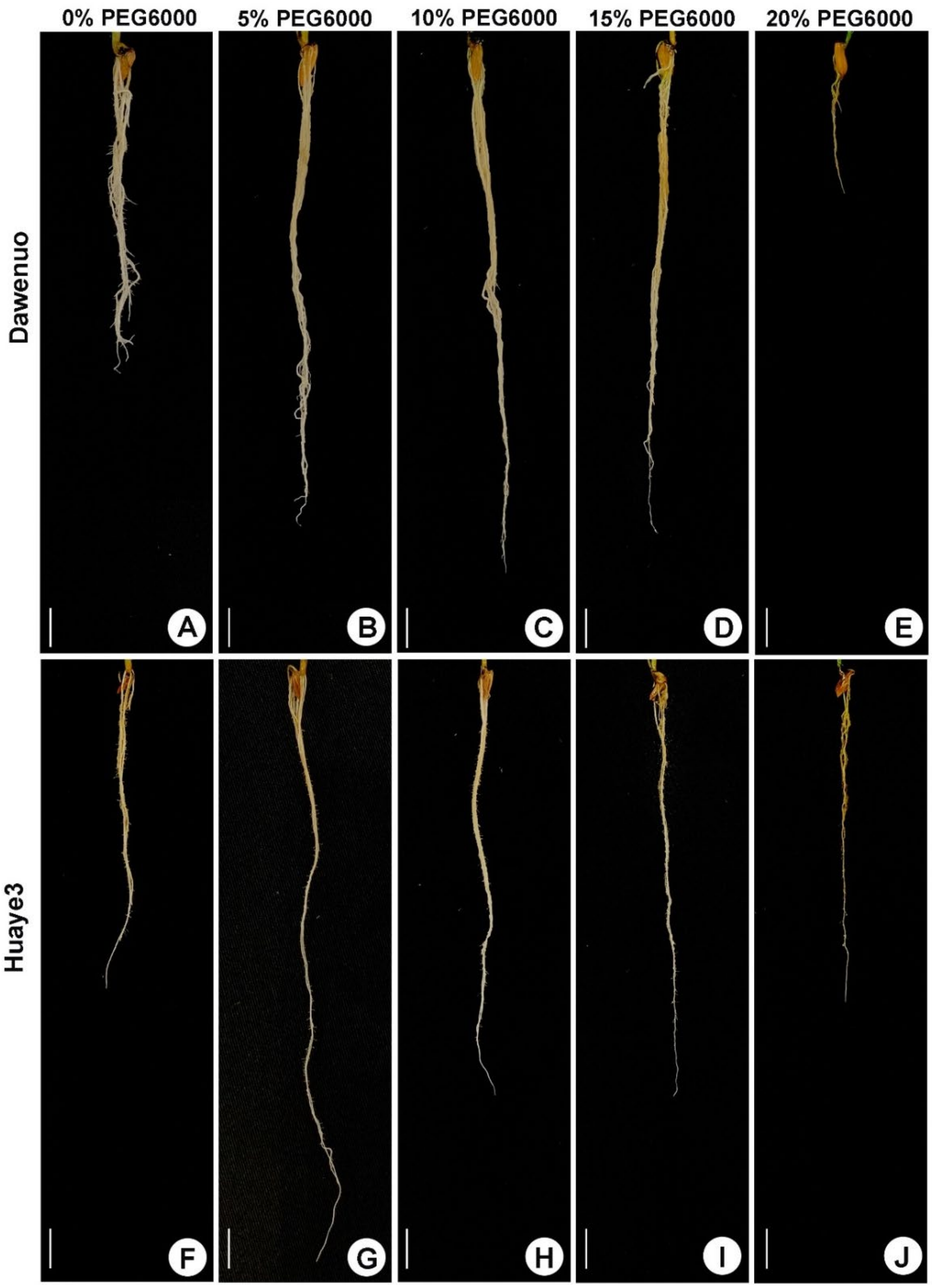

Figure S3. Comparison of the roots of Dawennuo and Huaye3 on the 30th day of growth under different PEG6000 concentration treatments

(A) The roots of Dawennuo on the 30th day with no PEG6000, CK. (B) The roots of Dawennuo on the 30th day under 5% PEG6000. (C) The roots of Dawennuo on the 30th day under 10% PEG6000. (D) The roots of Dawennuo on the 30th day under 15% PEG6000. (E) The roots of Dawennuo on the 30th day under 20% PEG6000. (F) The roots of Huaye3 on the 30th day with no PEG6000, CK. (G) The roots of Huaye3 on the 30th day under 5% PEG6000. (H) The roots of Huaye3 on the 30th day under 10% PEG6000. (I) The roots of Huaye3 on the 30th day under 15% PEG6000. (J) The roots of Huaye3 on the 30th day under 20% PEG6000. Bars=1 cm.

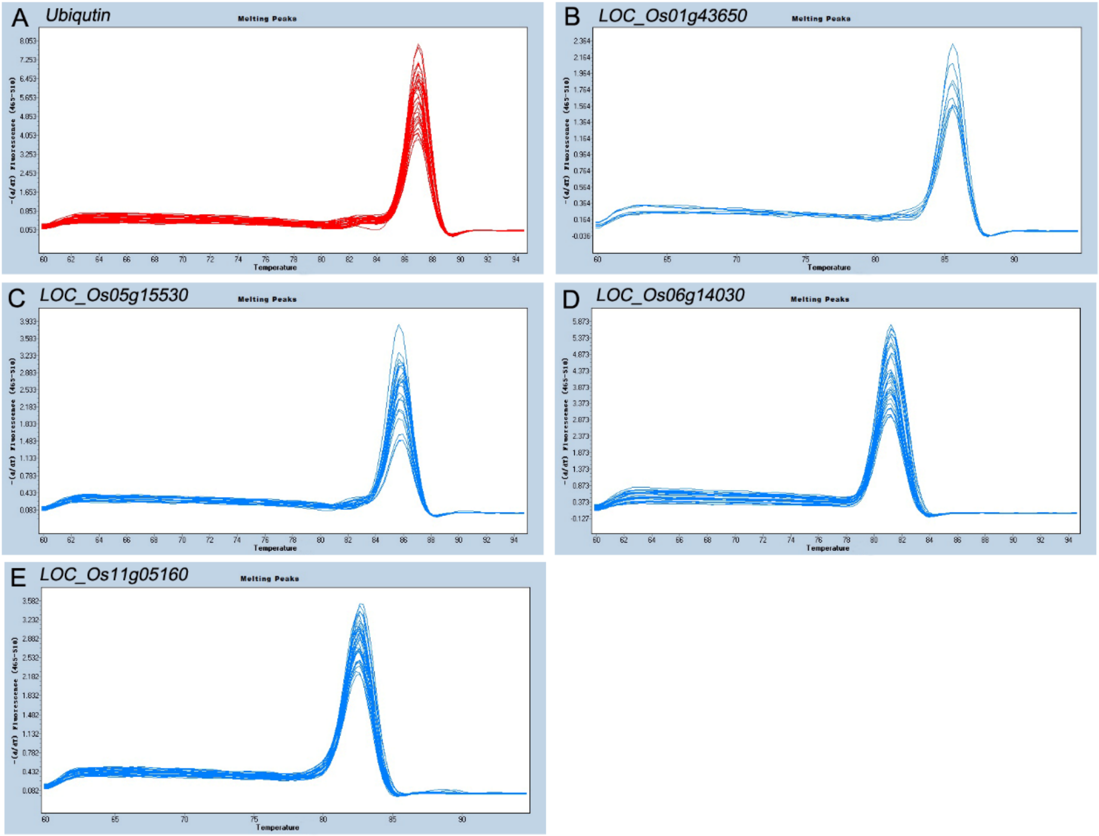

Figure S4. Melting curves of five candidate genes used for RT-qPCR analysis

(A) Melting curve of *Ubiqutin* gene show single peaks. (B) Melting curve of *LOC_Os01g43650* gene show single peaks. (C) Melting curve of *LOC_Os05g15530* gene show single peaks. (D) Melting curve of *LOC_Os06g14030* gene show single peaks. (E) Melting curve of *LOC_Os11g05160* gene show single peaks.
